# Holding Out for a Model: Rhomboid Superfamily in Vertebrate Development and Disease

**DOI:** 10.1002/jcp.70094

**Published:** 2025-09-28

**Authors:** Saroj Gourkanti, Yazmin Munoz, Jacqueline Cheung, Rosa M. Chavez, Devanshi Agarwal, Taylor J. Schoen, Kristina Solorio‐Kirpichyan, Sonya E. Neal

**Affiliations:** ^1^ Department of Cell and Developmental Biology, School of Biological Sciences University of California San Diego San Diego California USA; ^2^ Howard Hughes Medical Institute Chevy Chase Maryland USA

**Keywords:** development, membrane, rhomboid proteins, vertebrate model

## Abstract

The rhomboid superfamily, comprising both proteases and pseudoproteases, has emerged as a central regulator of membrane biology, mediating diverse functions including protein quality control, signal transduction, trafficking, and more. While molecular mechanisms of rhomboid activity have been well‐characterized in invertebrate and cell‐based systems, their physiological role in vertebrate development remains limited and continues to evolve. Here, we review recent advances in cell culture systems and vertebrate models that uncover the developmental and disease‐relevant functions of rhomboid family members, including RHBDLs, iRhoms, PARL, and Derlins. We outline their roles in embryogenesis, tissue regeneration, neurodevelopment, and immune signaling, alongside their pathological involvement in cancer, neurodegeneration, and metabolic disorders. We also emphasize the limitations posed by early embryonic lethality in knockout models and advocate for tissue‐specific vertebrate models to dissect rhomboid‐dependent pathways in vivo. Understanding how rhomboid proteins coordinate developmental processes will not only reveal fundamental principles of membrane‐associated processes, but also open new avenues for therapeutic targeting in disease.

## 1 Introduction

**Figure 1:**
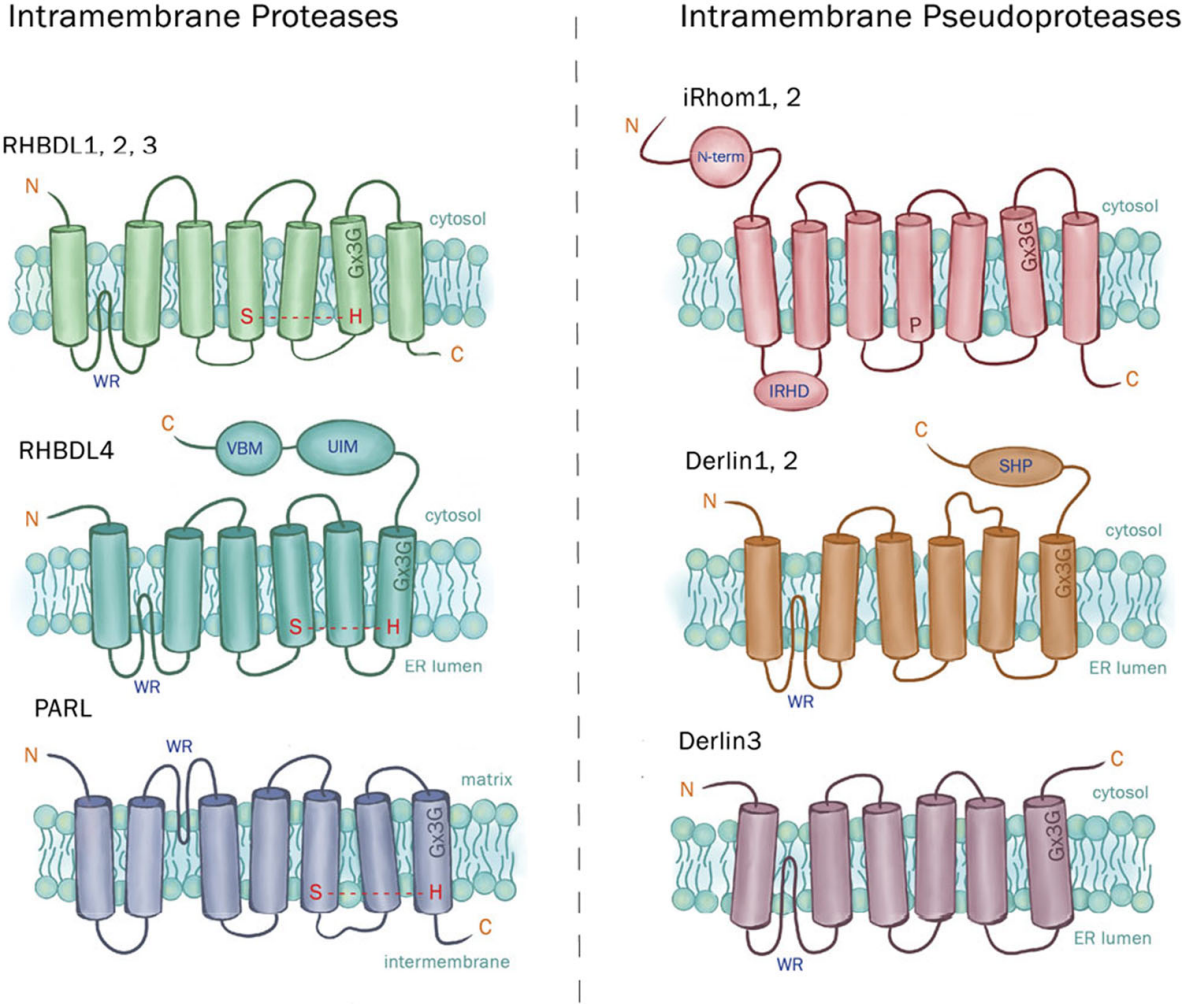
Depiction of members from the intramembrane protease and pseudoprotease subclass: RHBDL‐1, ‐2, ‐3, and ‐4, iRhom1&2, Derlin‐1, ‐2, and ‐3, and PARL, found in mammals, All rhomboid members diverged from a common ancestral rhomboid‐like core including six transmembrane helices (in blue), serine‐histidine catalytic dyad, and rhomboid motifs, WR and GxxxG, and evolutionarily diverged into the intramembrane and pseudoprotease subclass. Depicted in red are additional helices or domains that are unique to each rhomboid protein. The following domains are indicated: (VBM) valosin/p97‐binding motif (also known as SHP box), (UIM) ubiquitin‐interacting motif, (IRHD) iRhom homology domain, (UBA) ubiquitin‐associated domain.

The rhomboid superfamily comprises a unique group of intramembranous proteases and pseudoproteases that act as key regulators in cellular homeostasis. Members are present across all kingdoms of life and share a common structural architecture: 6 or 7 transmembrane helices, a highly conserved tryptophan‐arginine (WR) motif, and a GxxxG motif (Koonin et al. 2003; Wang et al. 2006; Lemberg and Freeman 2007; Nejatfard et al. 2021; Bhaduri, Scott et al. 2023). These proteins were first discovered in *Drosophila*, where a mutant screen identified Rhomboid‐1 mutants having a “pointy head” phenotype (Mayer and Nüsslein‐Volhard 1988). The name of this superfamily originates from the phenotypic defects observed in this early study and subsequent studies identified them as the first class of intramembranous serine proteases. Catalytically active members possess a conserved serine‐histidine (SH) dyad, enabling them to cleave substrates within the lipid bilayer (Urban et al. 2001; Lemberg et al. 2005; Urban and Wolfe 2005; Wu et al. 2006), whereas pseudoproteases lack this proteolytic activity, but are just as biologically important (Kandel and Neal 2020). These enzymes localize to various membrane‐bound compartments and possess unique substrate repertoires (Freeman 2014; Düsterhöft et al. 2017; Kandel and Neal 2020), highlighting the specific intracellular niches and functions of rhomboid‐like proteins (Figure 1).

**Figure 2:**
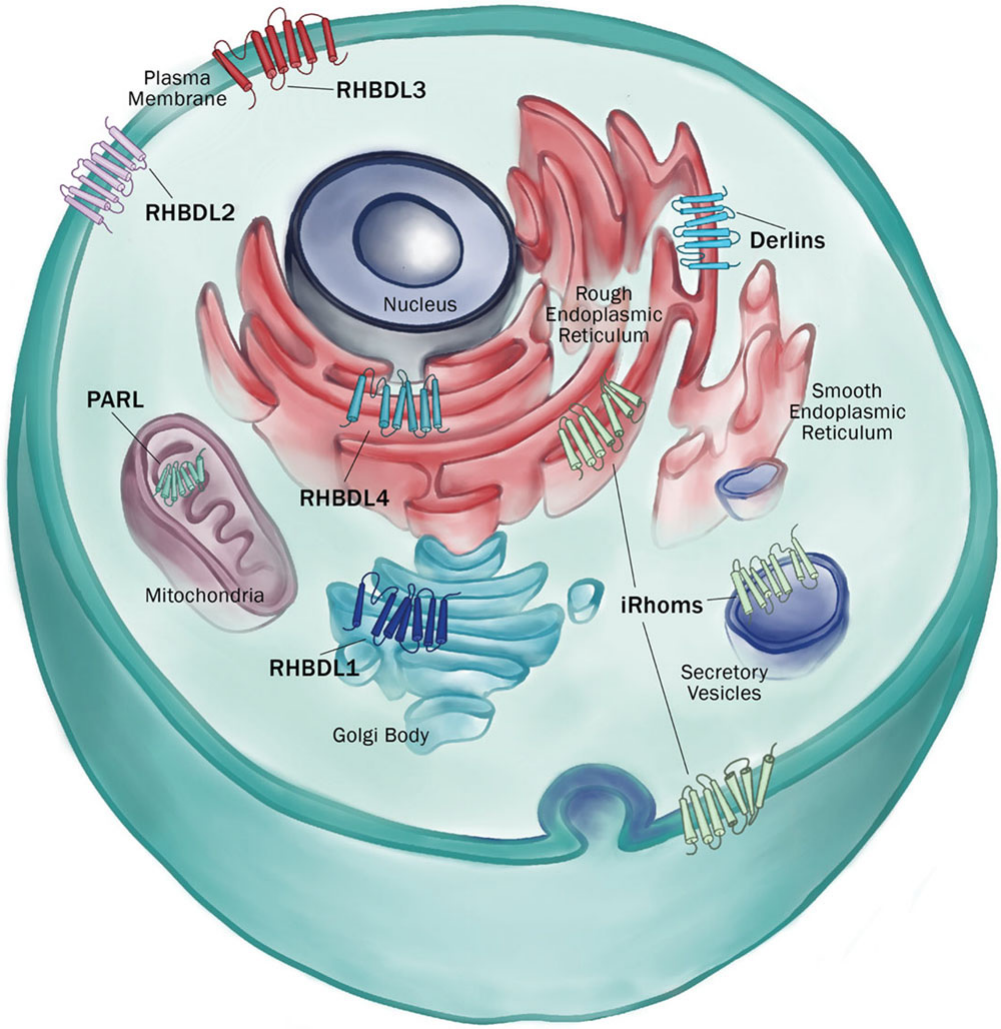
Depiction of subcellular localization of rhomboid superfamily members.

Mammalian rhomboid proteases consist of RHBDL1‐4 and mitochondrial integral membrane protein presenilin‐associated rhomboid like (PARL), while rhomboid pseudoproteases consists of iRhom and Derlin subclasses (Greenblatt et al. 2011; Zettl et al. 2011) (Figure 2). Additional rhomboid‐like pseudoproteases, such as UBAC2, RHBDD2, RHBDD3, and TMEM115, have also been characterized, are reviewed in detail elsewhere (Düsterhöft et al. 2017; Yamazoe et al. 2017; Adrain and Cavadas 2020). While in vitro studies have shed light on their cellular functions, the physiological roles of these proteins in vertebrate systems are only beginning to be uncovered, with much still to be learned.

**Figure 3:**
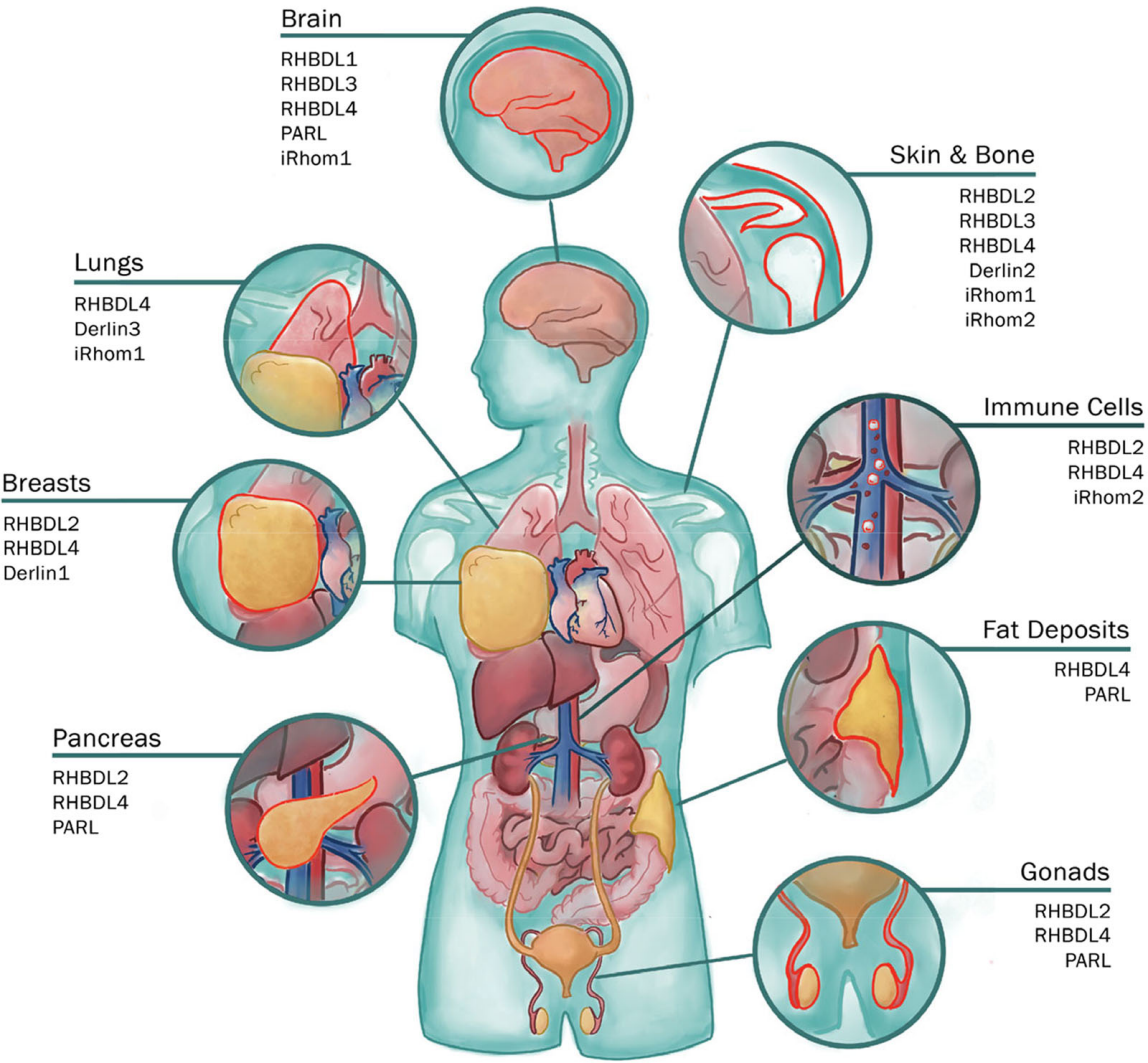
Schematic illustration showing various rhomboid family proteins, including RHBDL1–4, PARL, iRhom1/2, and Derlin proteins (Derlin1–3) implicated to function across various human tissues. Each inset highlights a specific organ or tissue and lists rhomboid‐related proteins with documented functional roles in that region, based on experimental evidence.

Studies in tissue culture and yeast have revealed the molecular mechanisms through which rhomboid‐like proteins maintain homeostasis and cellular interactions. These proteins localize to various membrane‐bound compartments, including the ER, Golgi, endosomes, mitochondria, and plasma membrane and are increasingly recognized as key regulators of vast membrane‐related processes such as proteostasis, lipid homeostasis, and intercellular signaling (Nejatfard et al. 2021; Greenblatt et al. 2011; Baldridge and Rapoport 2016). Although in vitro studies in tissue culture and yeast have uncovered key molecular functions of rhomboid proteins such as RHBDL4 and Derlins in ER protein quality control (Fleig et al. 2012) and RHBDL2 in ectodomain shedding (Adrain et al. 2011; Cheng et al. 2014; Koch et al. 2021), their physiological roles in whole organisms remain poorly understood. Studies investigating the function of various members in the rhomboid superfamily in vertebrate models are still in their early stages. Knockout attempts in mammals have led to viable mouse models for RHBDL4 (Han et al. 2023) and iRhom2 (Dulloo et al. 2019), but attempts for others have resulted in embryonic lethality (International Mouse Phenotyping Consortium, mousephenotype.org nd; Lastun et al. 2016). Nonetheless, recent advances have laid the groundwork for the further investigation of the wide range of developmental and disease roles of the rhomboid superfamily (Table 1 and Figure 3 summarize the diverse biological, physiological, and disease‐related functions of the rhomboid superfamily).

**Table 1:** Rhomboid proteins and their physiological and pathological roles, substrates, and pathways. Phenotypes described in the table have been identified from three categories: In vitro (tested in bacteria, yeast, tissue culture or patient samples), in vivo (tested in vertebrate animal models), or bioinformatics (potential role identified from sequencing databanks or phenotypic screen).

Rhomboid proteins	Main Physiological and pathological roles	Associated substrates	Molecular pathways	Published models	References
RHBDL2	Skin Regeneration (in vitro, in vivo)	Thrombomodulin, Spint1		None, embryonic lethality observed in mammalian knockouts	Cheng et al. (2011); Johnson et al. (2017)
	Bone Development and Homeostasis (in vitro, in vivo)	Thrombomodulin	IL11R signaling		Koch et al. (2021); Chen et al. (2020); Kespohl et al. (2021); Lokau et al. (2022); Kang et al. (2023)
	Female Fertility and Menopause (bioinformatics)	Unknown			Iager et al. (2013); Shi et al. (2016); Chang et al. (2021)
	Cancer (Pancreatic, breast) (in vitro, in vivo)		Notch signaling		Cheng et al. (2014); Chen, Cai et al. (2022); Qiao et al. (2021)
RHBDL4	Cancer (Lung, colorectal, gastric, and pancreatic) (in vitro, in vivo)	ZEB1/PI3K/AKT, Wnt/B‐catenin, EGFR, AKT/B‐catenin	ZEB1/PI3K/AKT, Wnt/B‐catenin, EGFR, AKT/B‐catenin	RHBDL4 mouse xenograft	Niu et al. (2019); Xu et al. (2021); Jiang et al. (2022); Wang et al. (2020); Yang et al. (2023)
	Cancer (glioblastoma, breast, ovarian, non small cell lung, colorectal) (in vitro, in vivo)		miRNA‐211‐3p, miR‐138, miR‐5195‐3p, miR‐294, mi145‐5p regulation		Zhao et al. (2019); Niu et al. (2019); Chen, Cai et al. (2022); Wang et al. (2023); Wang et al. (2020); Roshani et al. (2023)
	Alzheimer's disease (in vitro, in vivo)	amyloid precursor protein (APP)		Homozygous R4^‐/‐^ mouse	Paschkowsky et al. (2016); Penalva (2024)
	Charcot–Marie–Tooth disease (in vitro)	MPZ‐L170R			Fleig et al. (2012)
	Kawasaki disease and inflammation (in vivo, bioinformatics)	TMED7	TLR4 pathway	Homozygous R4^‐/‐^ mouse	Knopf et al. (2024)
	Lipid homeostasis (in vitro, in vivo)	SREBP‐1c		RHBDL4 CRISPR KO Mouse	Han et al. (2023); Shibuya et al. (2022)
	Spermatogenesis, and sex determination (bioinformatics)	Unknown			Wang, Hua et al. (2008); Tao et al. (2021)
	Growth, development, and apoptosis (in vitro)	tumor suppressor activated pathway‐6 (TSAP6) and BIK	EGFR signaling		Wan et al. (2012); Song et al. (2015); Wang, Hua et al. (2008); Wang et al. (2009); Tao et al. (2021)
	ER stress and homeostasis (in vitro, in vivo)		ER‐associated degradation (ERAD) pathway	Homozygous R4^‐/‐^ mouse	Kandel and Neal (2020); Freeman (2014); Lastun et al. (2022)
PARL	Adipogenesis (in vitro)	f‐PINK1	PARL‐PINK1‐Parkin pathway		Shiau et al. (2017)
	Mitochondrial biogenesis, apoptosis, and ferroptosis (in vitro)	Pgam5 (biogenesis), DIABLO (apoptosis), STARD7 (ferroptosis)	Wnt signaling, OPA1‐mediated cristae remodeling		Saita et al. (2017); Bernkopf et al. (2018); Liang and Jiang (2023)
	Spermatogenesis (in vivo)		Unknown but related to ferroptosis	full KO (*Parl* ^*‐/‐*^) mouse	Schumacher et al. (2024); Radaelli et al. (2023)
	Parkinson's disease (in vitro)	PINK1	PARL‐PINK1‐Parkin pathway		Shi et al. (2011)
	Dementia with lewy bodies (bioinformatics)	Unknown			Wüst et al. (2016)
	Alzheimer's disease (in vitro)	Unknown			Kawamoto et al. (2020)
	Amyotrophic lateral sclerosis (ALS) (in vitro, in vivo)	PINK1			Liu et al. (2023)
	Diabetic cardiomyopathy (in vitro, in vivo)		L‐carnitine mediated microvascular regulation		Li et al. (2023)
	Pancreatic Cancer (in vitro, in vivo)		STOML2‐mediated chemosenstitivity		Qin et al. (2023)
	Leigh‐like syndrome (in vivo)		Co‐enzyme Q depletion in electron transport chain	Conditional KO (*Parl* ^*lox/lox*^), full KO (*Parl* ^*‐/‐*^), and nervous system spec ific KO (*Parl* ^*L/L::*^ *Nes* ^*Cre*^) mouse	Spinazzi et al. (2019)
	Barth's syndrome (in vitro)		Cardiolipin regulaiton		Anzmann et al. (2021)
	Dopaminergic neurodegeneration (in vivo)	Unknown		Full KO (*parla* ^*‐/‐*^ *)* zebrafish	Merhi et al. (2021)
RHBDL3	Aging (bioinformatics)	Unknown		No characterized models	Kumar et al. (2013)
	Schizophrenia (bioinformatics)				Cerezo et al. (2025)
	Adolescent Idiopathic Scoliosis (bioinformatics)				
	Body height, chronotype measurement, blood pressure (bioinformatics)				
RHBDL1	Circadian rhythmicity (bioinformatics)	Unknown		None, embryonic lethality observed in mammalian knockouts	Zhang et al. (2020)
iRhom1	Cancer (breast, colorectal, liver, head and neck) (in vitro, in vivo)	ADAM17	EGFR, Wnt/β‐catenin signaling	iRhom1 siRNA in mouse	Luo et al. (2024); Zhou et al. (2014); Yuan et al. (2018)
	Childhood‐onset cardiomyopathy (bioinformatics)	Unknown			Al‐Hassnan et al. (2020)
	Bone development (in vivo)	ADAM17 Unknown	EGFR signaling (TGFɑ)	Chondrocyte‐specific deletion of iRhom1 in iRhom2 KO (*iRhom2* ^*‐/‐*^ *iRhom1* ^*fl/fl*^ *Col2a1‐cre*) mouse	Fang et al. (2020)
	Eyelid morphology (in vivo)	Presumed ADAM17	Presumed EGFR signaling	Epithelial‐specific deletion of iRhom1 in iRhom2 KO (*Rhbdf1* ^*flox/flox‐2.9kb*^ *Rhbdf2* ^*‐/‐*^ *KRT14‐Cre*) mouse	Erhardt et al. (2025)
	Endothelial development (in vivo)	Unknown		Endothelial‐specific deletion of iRhom1 in iRhom2 KO (*Rhbdf1* ^*flox/flox‐2.9kb*^ *Rhbdf2* ^*‐/‐*^ *Cdh5‐cre*) mouse	
iRhom2	Tylosis with esophageal cancer (TOC) (in vitro, in vivo, bioinformatics)	ADAM17	EGFR signaling (amphiregulin)	N‐terminal SNP in iRhom2 with presumed gain‐of function (*Rhbdf2* ^*P159L/P159L*^) mouse	Blaydon et al. (2012); Hosur et al. (2017); Saarinen et al. (2012); Mokoena et al. (2018)
	Skin and hair development (in vivo)			N‐terminal deletion in iRhom2 (*Rhbdf2* ^*cub/cub*^) mouse; N‐terminal deletion in iRhom2 (*Rhbdf2* ^*uncv/uncv*^) mouse	Hosur et al. (2014); Johnson et al. (2003); Siggs et al. (2014); Li et al. (1999); Leilei et al. (2014)
	Myocardial infarction (in vivo)		TNFɑ signaling	iRhom2 KO (*Rhbdf2* ^*‐/‐*^) mouse	Barnette et al. (2018)
	Lupus nephritis (in vivo)		TNFɑ, HB‐EGF/EGFR signaling		Qing et al. (2018)
	Liver disease (fibrosis, steatosis, alcohol induced damage) (in vivo)		TNFɑ, TNFR signaling		Liu et al. (2022); Sundaram et al. (2019)
	Viral infection (DNA and RNA viruses) (in vitro, in vivo)	STING, VISA	Interferon signaling		Luo et al. (2016); Luo et al. (2017)
	Bacterial infection (*Listeria monocytogenes*) (in vitro, in vivo)	ADAM17	TNFɑ signaling		McIlwain et al. (2012)
	Rheumatoid arthritis (in vitro, in vivo)				Issuree et al. (2013)
	Metabolic disease (hyperlipidemia, atherosclerosis) (in vivo)			iRhom2 KO in low‐density lipoprotein receptor KO (*LDLR* ^*‐/‐*^ *iRhom2* ^*‐/‐*^) mouse	Hannemann et al. (2022)
	Inflammatory bowel disease, colitis (in vitro, in vivo)		TNFɑ signaling (JAM‐A)		Giese et al. (2021)
	Alzheimer's disease (bioinformatics)	Unknown			De Jager et al. (2014)
Derlin‐1	Embryonic development (in vivo)	Unknown		Embryonic lethality observed in floxed KO (*Derl1* ^*f/f*^) mouse	Eura et al. (2012)
	Postnatal brain development (in vivo)			CNS specific deletion (*Derl1* ^*NesCre*^) mouse, neuron specific deletion (*Derl1* ^*CaMKIIαCre*^)	Sugiyama et al. (2021)
	Breast Cancer (in vitro)	TMEM63A	TOLLIP‐mediated autophagy		Wang, Hua et al. (2008)
	Muscle Invasive Bladder Cancer (in vitro, bioinformatics)		AKT/Bcl‐2 pathway		Dong et al. (2017)
Derlin‐2	Bone Development (in vivo)	Unknown		Floxed KO (*Derl2* ^*f/f*^) mouse, weaning issues observed	Dougan et al. (2011)
	Cholangiocarcinoma (in vitro, bioinformatics)				Liu et al. (2024)
	Diabetic nephropathy (in vivo)			Podocyte specific knockout (derlin‐2^∆podocyte^) mouse	Ren et al. (2018)
Derlin‐3	Lung adenocarcinoma (in vitro, bioinformatics)			Derlin‐3 full KO mice viable	Eura et al. (2012); Lin et al. (2022)

In this review, we highlight the emerging roles of rhomboid proteins in development and disease using cell culture and vertebrate model systems from the past 25 years. Along with in vivo studies that have associated rhomboid proteases & pseudoproteases with specific changes, we have compiled data from bioinformatics analyses that identify rhomboid proteases as novel factors contributing to disease. Throughout this review we use physiological and pathological subheadings for organizational clarity, but we view pathology as a continuum of dysregulation of rhomboid‐mediated pathways. In most cases, the same pathways that govern normal development and homeostasis explains the disease association we summarize. Finally, we introduce future perspectives on how rhomboid‐like proteins may be used as targets for therapies to attenuate the effects of biological stress using novel vertebrate knockout models.

## 2 Rhomboid Proteases

### 2.1 RHBDL2

#### 2.1.1 Physiological Role of RHBDL2

RHBDL2 is the mammalian homolog of *Drosophila* rhomboid‐1, the first rhomboid protease to be discovered (Urban et al. 2001). Localized primarily in the plasma membrane, RHBDL2 cleaves substrates that serve as key mediators of cellular signaling (Lohi et al. 2004). Its role in development primarily involves proliferation and migration of various cell types including keratinocytes and osteoblasts (Cheng et al. 2011; Chen et al. 2020). Proteomic studies identified substrates such as EGF, Spint1, and IL6R, suggesting RHBDL2 functions in epithelial homeostasis (Johnson et al. 2017). Accordingly, tissue culture studies on HaCaT cells demonstrated that RHBDL2‐mediated cleavage of its client substrate thrombomodulin (TM) induces cell proliferation and migration during regeneration (Cheng et al. 2011). Furthermore, RHBDL2 mediates cleavage of CLEC14A, where shedding of the lectin family protein induces sprouting angiogenesis (Noy et al. 2016). RHBDL2's involvement in bone development is becoming an area of growing interest. For instance, RHBDL2‐mediated cleavage of TM induces migration, proliferation, and mineralization of osteoblasts (Chen et al. 2020). RHBDL2 also potentially influences bone development as a regulatory sheddase in IL11R signaling (Koch et al. 2021; Kespohl et al. 2021; Lokau et al. 2022). Complementary experiments involving drug treatment against RHBDL2 in mice revealed impaired generation of new bone tissue following skull injury, suggesting these functions translate in vivo (Cheng et al. 2011; Chen et al. 2020). However, lack of a knockout vertebrate model presents a significant challenge in the field. Embryonic or postnatal lethality in mice models prevents researchers from studying RHBDL2's broader functions in development (International Mouse Phenotyping Consortium, mousephenotype.org nd). Nevertheless, results from systemic expression analyses are offering new insights on RHBDL2's systemic functions where it is postulated to be involved in osteoarthritis, reproductive success, and natural menopause (Iager et al. 2013; Shi et al. 2016; Chang et al. 2021). These studies touch on the role of RHBDL2 in different tissues at various stages of development. Future studies exploring tissue‐specific knockouts of RHBDL2 and identification of its substrate repertoire in vivo will offer deeper insights into RHBDL2‐dependent cell signaling across different cell types and tissues.

#### 2.1.2 Pathological Role of RHBDL2

RHBDL2 plays a major role in cancer progression and is a potential marker for various carcinomas. Indeed, patient sample analyses identified RHBDL2 as a prognostic marker in pancreatic cancer (PC), breast cancer, osteosarcoma, pancreatic ductal adenocarcinoma (PDAC), and colorectal cancer (Canzoneri et al. 2014; Rahman et al. 2020; Xiao et al. 2022; Chen et al. 2024). There are marked differences in RHBDL2's mechanism of action in different cancers. Increased RHBDL2 expression is correlated with PC incidence, proliferation, and metastasis in mouse xenografts through activation of the Notch signaling pathway (Chen, Wang et al. 2022). Analysis of patient samples in The Cancer Genome Atlas (TCGA) revealed that RHBDL2 contributes to high infiltration of cancer‐associated fibroblasts in osteosarcoma (Zhihao et al. 2023). Along with effects on migration and metastasis, induction of RHBDL2 prevents anoikis, or cell detachment, in breast and cervical cancer cell culture (Cheng et al. 2014). In contrast, RHBDL2 has been shown to delay cancer progression, with its upregulation being required for the spontaneous conversion of CD44−/CD24− breast cancer cells into CD44^+^/CD24− cancer stem cells in patient‐derived samples (Qiao et al. 2021). While mechanistic links to these different cancers are yet to be elucidated, it is likely that RHBDL2‐mediated cleavage of certain substrates such as CLCP1 may contribute to cancer progression (Schmoker et al. 2019). Beyond its role in cancer, RHBDL2 is also being explored in the context of other diseases, especially those affecting bone tissue. For instance, shedding of TM by RHBDL2 in chondrocytes decreases inflammation and osteoarthritis in mice (Kang et al. 2023), and suppression of RHBDL2‐mediated cleavage of TM induced by high glucose conditions prevents efficient bone healing in diabetic mice (Chen et al. 2020). Taken together, these studies offer just a glimpse into the broader role of RHBDL2 in disease. A vertebrate knockout model would provide a foundation for understanding how dysregulation of RHBDL2 drives pathologies such as cancer, regenerative defects, inflammation, and osteoarthritis.

### 2.2 RHBDL4

#### 2.2.1 Physiological Role of RHBDL4

The mammalian rhomboid protease RHBDL4, also called RHBDD1, is an intramembrane protease localized in the ER membrane. Tissue culture studies demonstrated that RHBDL4 primarily mitigates ER stress as a member of the ER‐associated degradation (ERAD) pathway (Freeman 2014). It achieves this by directly cleaving misfolded ER‐resident substrates, facilitating their removal from the ER membrane and subsequent degradation by the cytosolic proteasome (Fleig et al. 2012). While RHBDL4's role in ERAD highlights its importance in maintaining proteostasis (Knopf et al. 2020; Bock et al. 2022), identifying its substrates revealed how it also modulates diverse pathways beyond ER quality control. For example, it regulates ER sheet morphology by interacting with the ER stabilizing cytoskeletal linking membrane protein 63 (CLIMP‐63), further contributing to ER homeostasis (Lastun et al. 2022). RHBDL4 has also been shown to cleave TMED7, a p24 family cargo receptor involved in trafficking TLR4 (Toll‐like receptor 4). This cleavage downregulates TMED7, thereby dampening TLR4‐mediated inflammatory signaling (Knopf et al. 2024). RHBDL4 also targets and cleaves a p53‐inducible tumor suppressor‐activated pathway‐6 (TSAP6) (Wan et al. 2012). This cleavage promotes the secretion of proTGFα, a ligand that activates EGFR signaling, thereby linking RHBDL4 to pathways governing cell growth, proliferation, and differentiation (Song et al. 2015). Interestingly, another study suggested that RHBDL4 may instead facilitate the secretion of proTGFα without direct cleavage, highlighting alternative mechanisms by which this protease can modulate key regulatory signaling pathways (Wunderle et al. 2016). RHBDL4 has also been implicated in apoptosis through its cleavage of BIK, a proapoptotic tumor suppressor (Wang, Hua et al. 2008). Consistent with this finding, RHBDL4 knockdown in mouse GC‐1 stem cells increased apoptosis and impaired differentiation, highlighting its essential role in developmental processes (Wang et al. 2009). Although broadly expressed, RHBDL4 shows particularly high expression in the human testis, suggesting a role in spermatogenesis (Wang et al. 2009). Supporting this hypothesis, deleterious missense mutations in RHBDL4 were identified in sex‐patterned single‐nucleotide polymorphisms (SNPs) of Mozambique tilapia, pointing to potential functions in sex determination and germ cell development (Tao et al. 2021). Finally, RHBDL4 has been shown to regulate lipid metabolism. Epistatic analyses show high RHBDL4 expression in liver, pancreas, adipose‐subcutaneous, and adipose‐visceral tissues. RHBDL4 cleaves SREBP1, a transcription factor responsible for fatty acid and lipid synthesis and knocking out RHBDL4 in mice suppressed lipogenic gene expression (Han et al. 2023). In line with this, A232E/+ mice lacking functional RHBDL4 had impaired SREBP1 activation and fatty acid synthesis (Shibuya et al. 2022). Together, these findings underscore RHBDL4's contribution to various aspects of vertebrate development. However, despite recent advances, including the generation of an RHBDL4 knockout mouse model, the rhomboid protease remains poorly characterized at the vertebrate level. Further studies are needed to uncover its broader roles in cellular and developmental biology.

#### 2.2.2 Pathological Role of RHBDL4

RHBDL4 plays an important role in regulating cancer‐associated pathways. Its expression has been linked to a range of malignancies, including breast cancer, colorectal cancer, nonsmall cell lung cancer (NSCLC), ovarian cancer (OC), liver cancer, pancreatic adenocarcinoma, and glioma (Song et al. 2015; Ji‐Hua 2019; Zhao et al. 2019; Niu et al. 2019; Wu et al. 2021; Xu et al. 2021; Chen, Cai et al. 2022; Jiang et al. 2022; Jayathirtha et al. 2023; Wang et al. 2023). The most extensive investigations have focused on lung cancer, where RHBDL4 promotes oncogenic signaling via the ZEB1/PI3K/AKT and Wnt/B‐catenin pathways (Xu et al. 2021; Wang et al. 2020). Silencing of RHBDL4 suppresses these pathways, leading to reduced malignancy in NSCLC patient samples and mouse xenograft models, decreasing proliferation, invasion, and tumorigenesis (Wang et al. 2020). Similarly, downregulation of RHBDL4 impairs growth of pancreatic and gastric cancer in vivo, possibly via AKT/B‐catenin and Wnt/B‐catenin signaling (Jiang et al. 2022; Yang et al. 2023). Taken together, these results highlight how RHBDL4 is responsible for regulating cancer fates across multiple signaling pathways. Recently, increasing attention has been given to RHBDL4 regulation by various microRNAs (miRNAs) in cancer (Zhao et al. 2019; Niu et al. 2019; Chen, Cai et al. 2022; Wang et al. 2023; Wang et al. 2020; Koni et al. 2020; Wei et al. 2022; Roshani et al. 2023). For example, RHBDL4 has been identified as a target of miR‐5195‐3p, where its inhibition by the miRNA is associated with better prognosis in ovarian cancer patient samples (Wang et al. 2023). Furthermore, RHBDL4 knockdown in xenografted mice suppresses EGFR signaling and tumor growth in colorectal cancer, particularly when targeted by miR‐145‐5p (Niu et al. 2019; Koni et al. 2020; Wei et al. 2022; Roshani et al. 2023; Disoma et al. 2022). Understanding the interplay between RHBDL4 and miRNAs will be critical for assessing their utility as biomarkers for early cancer detection or therapeutic intervention.

Beyond cancer, RHBDL4 has been implicated in neurological and inflammatory diseases. It cleaves amyloid precursor protein (APP) in vitro, reducing secretion of Aβ peptide, the molecular driver of Alzheimer's disease (AD) (Paschkowsky et al. 2016). Studies in AD mouse models showed that absence of RHBDL4 rescued memory deficits (Penalva 2024). Furthermore, in the AD mouse models, the Wnt/β‐catenin pathway is differentially activated in RHBDL4‐deficient tissue, suggesting that RHBDL4 may act as a negative regulator of β‐catenin (Penalva et al. 2025). Interestingly, this contrasts with cancer models, where RHBDL4 instead promotes activation of the Wnt/β‐catenin pathway, highlighting its context‐dependent regulatory roles. RHBDL4 has also been implicated in Charcot‐Marie‐Tooth (CMT) disease, a degenerative nervous disorder characterized by weakened muscles and sensation. Cell‐based cleavage assays in HEK293T cells showed that RHBDL4 cleaves the pathogenic MPZ‐L170R, a mutant form of myelin protein zero linked to CMT (Fleig et al. 2012; Bhaduri et al. 2025). GWAS studies have linked loss‐of‐function mutations in RHBDL4 to Kawasaki disease, a pediatric inflammatory disorder marked by elevated cytokine levels. While its etiology remains unclear, RHBDL4 may influence disease pathogenesis through its role in innate immune regulation (Knopf et al. 2024; Buda et al. 2021).

### 2.3 PARL

#### 2.3.1 Physiological Role of PARL

PARL is a rhomboid protease present in the mitochondrial inner membrane (McQuibban et al. 2003; Jeyaraju et al. 2006; Hill and Pellegrini 2010; Chan and McQuibban 2013). Like other rhomboid proteases, PARL regulates cell proliferation and development in a variety of cell types such as neurons (Merhi et al. 2021), liver cells (Sekine et al. 2012), and cultured HEK293 and HeLa cells (Jeyaraju et al. 2006) by cleaving key signaling proteins within the mitochondrial inner membrane. It plays a central role in the regulation of the PINK1/Parkin pathway, which is not only implicated in Parkinson's disease (discussed more in depth under pathological roles below), but also in adipocyte differentiation. Silencing of PARL disrupts adipogenesis due to incomplete cleavage of f‐PINK1, as shown in both cultured adipocytes and animal models (Shiau et al. 2017). PARL also regulates mitochondrial biogenesis and apoptosis through cleavage of substrates such as Pgam5 and DIABLO, thereby promoting Wnt signaling for biogenesis and OPA1‐mediated cristae remodeling for apoptosis (Saita et al. 2017; Bernkopf et al. 2018). A PARL knockout in mouse brains leads to increased susceptibility to ferroptosis due to impaired cleavage of STARD7. This affects the biosynthesis and distribution of co‐enzyme Q, a key component of the electron transport chain along the mitochondrial inner membrane (Liang and Jiang 2023). Beyond its role in mitochondrial function and biogenesis, PARL is required for spermatogenesis. In 8‐week‐old PARL KO mice, testes exhibit a complete loss of HSD17B3, an ER protein required for the final step of testosterone biosynthesis (Schumacher et al. 2024). Additionally, these KO mice display premature ferroptosis in spermatocytes, further connecting PARL's mitochondria functions to physiological outcomes (Radaelli et al. 2023). Collectively, these findings highlight PARL's involvement in numerous developmental pathways and underscore the need for further studies to fully delineate its roles in physiological development.

#### 2.3.2 Pathological Role of PARL

PARL is a key regulator of the PINK1‐Parkin pathway, a major axis associated with the pathogenesis of Parkinson's disease (PD) (Jin et al. 2010; Meissner et al. 2015; Okatsu et al. 2015; Quinn et al. 2020). Over the past decade, mechanistic studies have shed light on the role of PARL in this process. Specifically, PARL regulates proteolytic processing of the kinase PINK1, which is required for the recruitment of the E3 ligase PARKIN in mouse embryonic fibroblasts and supported by patient‐derived PD patient samples (Shi et al. 2011). Notably, PARL missense mutations have been identified in individuals with PD, positioning it as a potential therapeutic target (Wüst et al. 2016). Similarly, in zebrafish models, loss of *parla* through CRISPR manipulations resulted in a significant loss of dopaminergic neurons especially in the olfactory bulb, confirming PARL's involvement in proper functioning of the brain in zebrafish (Merhi et al. 2021). Additionally, PARL accumulates in cortical‐type and brainstem Lewy bodies in samples collected from patients suffering from PD or dementia with Lewy bodies (DLB) (Kawamoto et al. 2020). While the exact role of PARL in the pathogenesis of DLB remains to be studied, it is hypothesized that accumulation of PARL along with antiapoptotic protein HAX‐1 contributes to the formation of Lewy bodies (Kawamoto et al. 2020). Beyond PD and DLB, PARL dysfunction has been implicated in amyotrophic lateral sclerosis (ALS). In brain tissues from ALS patients and ALS mouse models, disruption of mitophagy flux involving PARL, PINK1 and the mitochondrial cristae resident protein CHCHD10 was observed (Liu et al. 2023). A genome‐wide survival analysis also identified PARL as a locus for clinical progression and pathogenesis in AD (Chen et al. 2023).

Besides neurodegeneration, PARL defects have also been identified in other diseases. PARL/PINK1‐mediated mitophagy was shown to be regulated by L‐carnitine and STOML2 to alleviate cardiac microvascular dysfunction in diabetic cardiomyopathy in mice, and chemosensitivity in PC cells and mouse models, respectively (Li et al. 2023; Qin et al. 2023). Mouse models with PARL deficiency also display defects in Complex III of the electron transport chain, co‐enzyme Q depletion (as discussed under physiological roles above), and symptoms of Leigh‐like syndrome (Spinazzi et al. 2019). Interestingly, PARL may function independently of PINK1 in certain contexts. In cells lacking tafazzin, a mitochondrial membrane protein, PARL expression was upregulated, suggesting a role in cardiolipin regulation and a possible link to Barth syndrome (Anzmann et al. 2021). Together, these findings highlight PARL's multifaceted involvement in the pathogenesis of neurodegenerative diseases, metabolic syndromes, and cancer. Its centrality in mitophagy and mitochondrial quality control underscores its therapeutic potential, although more research is needed to fully elucidate its role across diverse disease states.

### 2.4 RHBDL1 and RHBDL3

#### 2.4.1 Physiological Role of RHBDL1 and RHBDL3

RHBDL1 and RHBDL3 remain the most enigmatic proteases of the rhomboid superfamily, with no characterized functions despite high conservation and lethal phenotypes in mouse knockout models (International Mouse Phenotyping Consortium, mousephenotype.org nd). Despite sharing 50% sequence identity, these rhomboid‐like proteins exhibit distinct cellular localizations. RHBDL1 appears to be restricted to the Golgi apparatus, whereas RHBDL3 (previously known as ventrhoid) is widely distributed in the secretory pathway, localized in the plasma membrane and endosomal compartments (Lohi et al. 2004). Although both have distinct cellular localizations, they exhibit overlapping expression patterns in neurons and various brain regions, suggesting a potential shared role in neuronal processes (Strisovsky 2016). Together with their shared sequence homology, this suggests potential convergence in neurological functions. Notably, RHBDL3 possesses EF‐hand motifs enriched in calcium‐binding residues within its cytoplasmic N‐terminal domain, indicating that its activity may be regulated by calcium signaling (Lastun et al. 2016; Baker and Urban 2015). The limited research on RHBDL3 and RHBDL1 highlights the need to define their functions, as a deeper understanding of these proteases could offer important insights into neuronal development and function.

#### 2.4.2 Pathological Role of RHBDL1 and RHBDL3

The roles of RHBDL1 and RHBDL3 in disease remain poorly understood due to limited research. However, their established importance in development suggests that dysregulation of these proteases may contribute to human pathologies. Notably, RHBDL3 has been implicated in aging, showing one of the strongest correlations with chronological age (Kumar et al. 2013), and may serve as a potential biomarker. RHBDL3 is further implicated in a range of conditions, with SNPs associated with schizophrenia, body height, adolescent idiopathic scoliosis, chronotype measurement, and blood pressure (Cerezo et al. 2025). Its high expression in the brain aligns with its potential involvement in neuron‐related pathologies. Similarly, RHBDL1 has been proposed as a genetic determinant of circadian rhythmicity and responsiveness to light‐dark cycles, reinforcing the notion that, like RHBDL3, it is involved in neuropathological processes (Zhang et al. 2020). While further studies are needed to clarify their specific functions, current data point to RHBDL1 and RHBDL3 as candidate regulators in diverse aspects of human health and disease.

## 3 Rhomboid Pseudoproteases

### 3.1 iRhoms

#### 3.1.1 Physiological Role of iRhoms

iRhom1 (RHBDF1) and iRhom2 (RHBDF2) are rhomboid‐like proteins that lack proteolytic activity, classifying them as rhomboid pseudoproteases. Despite their classification as pseudoproteases, both iRhom1 and iRhom2 play critical roles in regulating growth factor and inflammatory signaling by facilitating trafficking, maturation, and activity of client proteins throughout the cell, as demonstrated in both in vitro systems and animal models (Zettl et al. 2011; Adrain et al. 2012; Christova et al. 2013; Lee et al. 2015; Li et al. 2015; Grieve et al. 2017; Künzel et al. 2018). In mammals, iRhom1 is widely expressed, whereas iRhom2 expression is mostly restricted to immune cells and epithelial tissues (Christova et al. 2013; Künzel et al. 2018; Blaydon et al. 2012; McIlwain et al. 2012; Tüshaus et al. 2021). iRhoms share multiple cellular functions, including regulation of inflammatory and growth factor signaling molecules (Christova et al. 2013; Li et al. 2015). For instance, the cell surface metalloprotease ADAM17 requires iRhoms and is responsible for shedding the major pro‐inflammatory cytokine tumor necrosis factor alpha (TNF‐α) and epidermal growth factor receptor (EGFR) ligands (Adrain et al. 2012; Li et al. 2015; McIlwain et al. 2012).

iRhoms are essential for embryonic development; mice lacking both iRhoms exhibit perinatal lethality and multiorgan pathology, closely resembling phenotypes observed in mice lacking ADAM17 (Christova et al. 2013; Li et al. 2015; Veit et al. 2019; Hosur et al. 2020). Insights into the distinct physiological functions of iRhom1 and iRhom2 can be gleaned from studies of single loss‐of‐function and gain‐of‐function (GOF) animal models. iRhom2‐deficient mice are consistently healthy and display no developmental defects (Christova et al. 2013; Li et al. 2015). However, defining a clear developmental role for iRhom1has been complicated by conflicting phenotypes of iRhom1‐deficient mice. The first reported iRhom1‐deficient mice died within weeks of birth (Christova et al. 2013). These mice lacked the entire iRhom1 coding sequence (exons 2‐18) and display multiorgan pathologies including brain hemorrhage, growth deficiency, and cardiac defects, phenotypes resembling iRhom1/iRhom2 double knockout mice. In a conflicting report, mice lacking exons 4‐11 of iRhom1 were found to be healthy, viable, and fertile (Li et al. 2015). Subsequent work revealed that the mutation strategy used in Li et al. (2015) yielded truncated yet functional iRhom1 protein variants through alternative promoter usage and exon skipping, and therefore these mice are not entirely iRhom1‐deficient (Hosur et al. 2020). Furthermore, this mutant allele was sufficient to rescue lethality of iRhom1 and iRhom2 double knockout mice (Hosur et al. 2020; Burzenski et al. 2021), suggesting that these iRhom1 variants maintained essential functions. These results support initial reports that iRhom1 is indeed essential and that iRhom2 is unable to fully compensate during development. Differences in mouse mutant phenotypes between iRhom1 and iRhom2 may be attributed to differences in tissue expression patterns. Conditional knockout and tissue‐specific rescue approaches can further define specific roles for iRhoms in development. Chondrocyte‐specific deletion of iRhom1 in an iRhom2 KO mice background revealed a role for iRhom1 in bone development (Fang et al. 2020). Recent advancements with tissue‐specific Cre drivers revealed essential roles for iRhom1 in keratinocytes and endothelial cells in mice (Erhardt et al. 2025). Loss of iRhom1 in keratinocytes in an iRhom2‐deficient background (*Rhbdf1* ^*flox/flox‐2.9kb*^ *Rhbdf2* ^*‐/‐*^ *KRT14‐cre)* resulted in the “open eyelid birth” phenotype that is typical of iRhom1/iRhom2 double knockout animals, indicating that expression of iRhom1 and iRhom2 in keratinocytes regulates this defect. Additionally, endothelial‐specific deletion of iRhom1 using a Cdh5‐cre driver in iRhom2 KO mice (*Rhbdf1* ^*flox/flox‐2.9kb*^ *Rhbdf2* ^*‐/‐*^ *Cdh5‐cre*) causes *in utero* lethality, revealing that iRhom1 function in endothelial cells is essential for survival. It is also possible that unique substrate specificities contribute to selective physiological roles of these proteins. For example, gain‐of‐function mutations in the N‐terminus of iRhom2, but not iRhom1, are associated with alterations in skin and hair (Blaydon et al. 2012; Hosur et al. 2014; Hosur et al. 2017). In a model of the human disease Tylosis with esophageal cancer (TOC) (Rhbdf2‐P159L), mice are born hairless and later develop thin, curly coats (Hosur et al. 2017). Rhbdf2‐P159L mice also exhibit accelerated wound healing, which is attributed to increased secretion of the EGFR ligand amphiregulin (AREG) (Hosur et al. 2017). Similar phenotypes are observed in the iRhom2 variant Rhbdf2‐*cub* (*curly bare*), which lacks the first 268 amino acids of the N‐terminus, however, reports vary on whether the *cub* mutation represents a gain‐of‐function mutation and whether increased AREG secretion is the driving mechanism of defective hair development (Hosur et al. 2014; Johnson et al. 2003; Siggs et al. 2014). The distinct developmental phenotypes of iRhom mutant animals highlight the unique roles of these proteins in vivo. Future work addressing tissue‐ and ligand‐specific functions of iRhoms should provide more detail on their role in vertebrate biology.

#### 3.1.2 Pathological Role of iRhoms

iRhoms are implicated in various human diseases including cancer, neurodegeneration, autoimmunity, infection, and inflammatory digestive conditions (see Table 1). iRhom expression must be tightly regulated to maintain organismal health, as both increased and decreased activity contributes to disease. Overexpression of iRhom1 and iRhom2 has been linked to various cancers and inflammatory diseases. As indicated in the previous section, naturally occurring activating mutations in the N‐terminus of iRhom2 (I86T, P189L, D188N), which enhance EGFR signaling, are known drivers of Tylosis with esophageal cancer (TOC) in people (Blaydon et al. 2012; Saarinen et al. 2012; Mokoena et al. 2018). These mutations are unique to iRhom2, as activating mutations in iRhom1 have not been reported or associated with human disease. Furthermore, a poor prognosis is associated with increased expression of both iRhom1 or 2 in colorectal, cervical, breast, pancreatic, head/neck, and liver cancers (Zou et al. 2009; Luo et al. 2024). Given these findings, iRhoms are gaining attention as potential therapeutic targets. Inhibition of iRhom1 and thereby blocking subsequent ADAM17‐mediated shedding of CD44, enhanced tumor uptake of CD44 directed chemotherapeutics and increased tumor clearance in multiple cancers in mice (Luo et al. 2024). There has been a major focus on iRhom2 in the context of inflammatory diseases, particularly TNFα‐driven pathologies (see Table 1). Upregulation of iRhom2, but not iRhom1, is found in people with irritable bowel disease, Crohn's disease, and ulcerative colitis (Giese et al. 2021). The association of iRhom2 with inflammatory gut diseases is inherently linked to its expression in leukocytes and epithelial cells. In a mouse model of acute colitis, iRhom2/ADAM17 activity drove damaging inflammation and impaired epithelial integrity through ADAM17 shedding of cell adhesion molecules (Giese et al. 2021). While decreasing iRhom2 activity may alleviate damage in hyper‐inflammatory conditions, there is also an important homeostatic role for iRhom2 in epithelial tissues. In the few reported cases of congenital iRhom2 mutations, patients with homozygous frameshift mutations in iRhom2 have loss of iRhom2 protein and impaired ADAM17 cleavage of TNF‐α and amphiregulin. While otherwise healthy, these children experience recurrent respiratory infections and colitis (Kubo et al. 2022). These patients highlight that the beneficial or harmful contributions of iRhom2 during disease are largely context dependent. For example, iRhom2 plays opposing roles in distinct types of liver damage. During alcohol‐induced liver damage in mice, iRhom2‐driven release of TNF‐α contributed to oxidative stress and inflammation characteristics of the disease (Liu et al. 2022). However, in fibrotic liver disease, loss of iRhom2 protected mice from fibrosis due to decreased shedding of the TNF‐α receptor, TNFR, by ADAM17 in a specific population of liver cells (Sundaram et al. 2019). iRhom2 may also help to mediate the negative effects of diet‐induced inflammation. In conflicting reports, iRhom2 knockout animals were more (Badenes et al. 2020) or less (Skurski et al. 2020) resistant to insulin resistance and fat gain when fed a high‐fat diet. In another instance, loss of iRhom2 was protective against hyperlipidemia and reduced atherosclerotic lesion size in mice fed a Western diet (Hannemann et al. 2022). Taken together, these studies highlight the context‐dependent impacts of iRhom2 during inflammatory disease. In contrast to the wealth of data regarding iRhom2, we have far less knowledge of iRhom1 functions in disease. While total iRhom1 knockout causes prenatal lethality in mice (Christova et al. 2013; Hosur et al. 2020), the recent advancements in generating iRhom1 conditional knockouts (Erhardt et al. 2025) will undoubtedly provide opportunities for further investigation of iRhom1 in disease. In addition to a focus on developing tissue‐specific and conditional knockouts, exploring alternative vertebrate models like the zebrafish, will be of great value to the field.

### 3.2 Derlins

#### 3.2.1 Physiological Role of Derlins

The yeast derlin, Der1, was identified in a screen for mutants defective in ER‐associated degradation (Knop et al. 1996). The same group subsequently discovered an additional ER‐localized derlin, Dfm1 (Hitt and Wolf 2004), which was later characterized as a critical factor in ER homeostasis (Sato and Hampton 2006) and ER‐associated degradation of membrane substrates (Neal et al. 2018). In mammals, the yeast derlin homologs are Derlin‐1, Derlin‐2, and Derlin‐3. Primary sequence alignments show that Derlin‐1 shares 30% sequence identity with Derlin‐2 and ‐3, while Derlin‐2 and ‐3 share 70% sequence identity. RNA analysis of human tissues revealed that Derlin‐1 and Derlin‐2 are globally expressed in all tissues, while Derlin‐3 expression is limited to the pancreas, placenta, spleen, and small intestine (Oda et al. 2006). Derlins were first identified in 2004 by Ploegh and colleagues while investigating how human cytomegalovirus (HCMV) evades immune detection by promoting the degradation of class I major histocompatibility complex (Lilley and Ploegh 2004). In this study, they identified Derlin‐1, a 22 kDa transmembrane protein that associates with human cytomegalovirus‐encoded glycoprotein US11, and shares limited similarity with yeast protein Der1, known for its key role in dislocating ER misfolded proteins. They further characterized Derlin‐1 as a key player in the degradation of class I major histocompatibility complex proteins, thereby aiding viral propagation by avoiding detection by the immune system (Lilley and Ploegh 2004). Derlins are well‐characterized for the mechanistic role in ERAD, where they facilitate the retrotranslocation of misfolded protein across the ER membrane (Nejatfard et al. 2021; Oda et al. 2006; Lilley and Ploegh 2004; Ye et al. 2004). Notably, both yeast Derlins (Der1 and Dfm1) and mammalian Derlin (Derlin‐1) employed lipid‐thinning to facilitate substrates to move across the ER membrane for cytosolic degradation by the proteasome (Nejatfard et al. 2021). In addition, Derlins have been implicated in ERAD‐independent roles in regulating sphingolipid metabolism, underscoring their broader relevance in protein quality control and lipid homeostasis (Bhaduri, Scott et al. 2023). Despite their well‐characterized mechanistic roles, studies investigating the physiological functions in vertebrates remain limited, largely due to the lethality associated with their loss. While Derlin‐3 KO mice are viable and appear normal, global deletion of Derlin‐1 results in embryonic lethality at embryonic day 7 and 8 (Eura et al. 2012) and most Derlin‐2 KO mice die within 24 h of birth due to failure to feed (Dougan et al. 2011). Among the few Derlin‐2 deficient mice that survive to adulthood, skeletal abnormalities have been reported due to intracellular accumulation of collagen matrix proteins in the ER and increased apoptosis of chondrocytes (Dougan et al. 2011). More recently, central nervous system‐specific deletion of Derlin‐1 and Derlin‐2 resulted in impaired postnatal brain development and motor function, which may stem from delayed dendritic outgrowth linked to reduced SREBP2‐mediated cholesterol biosynthesis (Sugiyama et al. 2021). In summary, Derlins are critical for embryonic viability, skeletal and neural development, and motor function in mice. Expanding the use of conditional Derlin knockout models in mice and other vertebrate systems will be crucial for dissecting their tissue‐specific roles at both early and late stages of the developmental process.

#### 3.2.2 Pathological Roles of Derlins

Derlins are frequently overexpressed in a range of cancer types, where they help tumor cells adapt to cytotoxic stress. As tumors grow rapidly, they trigger intrinsic stress pathways, such as the unfolded protein response (UPR), to maintain proteostasis and support survival (Wang and Kaufman 2014). Derlin‐1 is notably upregulated in human breast cancer tissues, likely as an adaptive response to ER stress that shields cancer cells from ER stress‐induced apoptosis (Wang, Guan et al. 2008). In triple‐negative breast cancer (TNBC), a recent study identified transmembrane protein 63 A (TMEM63A) as a novel oncogene that stabilizes Derlin‐1 by blocking its degradation via Toll interacting protein (TOLLIP)‐mediated autophagy (Zhang et al. 2022). Notably, pharmacological inhibition of valosin containing protein (VCP) or depleting Derlin‐1 in mice reduces TMEM63A‐mediated triple negative breast cancer progression and metastasis (Zhang et al. 2022). In addition to its role in breast cancer, Derlin‐1 is overexpressed in muscle invasive bladder cancer, where its elevated expression correlates with poor prognosis (Dong et al. 2017). Mechanistic studies suggest Derlin‐1 promotes chemoresistance through activation of the AKT/Bcl‐2 survival pathway (Dong et al. 2017). Similar overexpression patterns and potential functional contributions have also been observed for Derlin‐2 (Liu et al. 2024) and Derlin‐3 (Lin et al. 2022) in other cancer contexts, suggesting a broader oncogenic role for the Derlin family. RNA sequencing data from the TCGA show that Derlin‐2 mRNA is elevated in patients with cholangiocarcinoma (CHOL), where its upregulation correlates with worse survival outcomes (Liu et al. 2024). Functionally, Derlin‐2 contributes to chemotherapy resistance in CHOL cells similarly to Derlin‐1, supporting its potential as a therapeutic target to limit CHOL progression (Liu et al. 2024). In a separate context, Derlin‐2 also plays a protective role in kidney disease. Podocyte‐specific Derlin‐2 knockout mice are more susceptible to adriamycin‐induced glomerulosclerosis (Ren et al. 2018). Additionally, Derlin‐2 protein levels are elevated in glomerular podocytes in mouse models and patients with diabetic nephropathy, suggesting a renoprotective role during ER stress (Ren et al. 2018). Higher levels of Derlin‐3 protein were detected in human patient lung adenocarcinoma tissues compared to adjacent tissues, and patients with higher levels of Derlin‐3 also responded poorly to chemo and immunotherapies, suggesting upregulation of Derlin‐3 may be a potential indicator for poor sensitivity to therapeutics (Lin et al. 2022). Together, these findings highlight the relevance of Derlins in cancer progression and kidney disease, positioning them as a candidate for cancer therapeutics.

## 4 Conclusion

Research into the physiological and pathological roles of the rhomboid superfamily has rapidly expanded over the past 25 years. Their remarkable structural conservation and presence across all domains of life reflect their fundamental biological importance. While vertebrate model systems have begun to reveal their cellular functions and organismal relevance, most studies to date have focused on knockout phenotypes, leaving their precise mechanisms of action in multicellular contexts largely unexplored. In particular, the contribution of rhomboid proteins to coordinated cellular behaviors during vertebrate embryogenesis remains poorly understood. To bridge these gaps, studies across vertebrate systems provide powerful opportunities to dissect rhomboid‐dependent processes in vivo with high resolution. Given the limitations of mammalian systems, particularly early embryonic lethality, other vertebrate models offer valuable opportunities to explore the physiological relevance of rhomboid proteases (Bhaduri, Aguayo et al. 2023; Bergbold and Lemberg 2013). For example, zebrafish provide unique advantages due to their optical transparency, which enables high‐resolution tracking of embryogenesis and development. In addition, the breadth of available genetic tools and disease‐relevant assays make zebrafish a versatile system for dissecting rhomboid function in diverse contexts, including biochemical pathways and drug discovery. Ultimately, advancing our understanding of the rhomboid superfamily in disease‐relevant settings will enable their strategic targeting in therapeutic development.

## Author Contributions

The review was conceptualized by Saroj Gourkanti and Yazmin Munoz. The original draft was prepared by all authors, with all authors also contributing to the review and editing process. Figures were prepared by Jacqueline Cheung.

## Conflicts of Interest

The authors declare no conflicts of interest.

## Data Availability

Data sharing not applicable to this article as no data sets were generated or analyzed.
